# MSP-4, an Antimicrobial Peptide, Induces Apoptosis via Activation of Extrinsic Fas/FasL- and Intrinsic Mitochondria-Mediated Pathways in One Osteosarcoma Cell Line

**DOI:** 10.3390/md16010008

**Published:** 2018-01-02

**Authors:** Hsiao-Mei Kuo, Chung-Chih Tseng, Nan-Fu Chen, Ming-Hong Tai, Han-Chun Hung, Chien-Wei Feng, Shu-Yu Cheng, Shi-Ying Huang, Yen-Hsuan Jean, Zhi-Hong Wen

**Affiliations:** 1 Center for Neuroscience, National Sun Yat-sen University, Kaohsiung 80424, Taiwan; Hsiaomeikuo@gmail.com (H.-M.K.); minghongtai@gmail.com (M.-H.T.); 2 Department of Marine Biotechnology and Resources, National Sun Yat-sen University, Kaohsiung 80424, Taiwan; caviton@gmail.com (C.-C.T.); hanchun25@gmail.com (H.-C.H.); qscjuejuejue@gmail.com (C.-W.F.); joygetit@gmail.com (S.-Y.C.); 3 Department of Dentisry, Zuoying Branch of Kaohsiung Armed Forces General Hospital, Kaohsiung 81357, Taiwan; 4 Department of Neurosurgery and Surgery, Kaohsiung Armed Forces General Hospital, Kaohsiung 80284, Taiwan; chen06688@gmail.com; 5 Department of Neurological Surgery, Tri-Service General Hospital, National Defense Medical Center, Taipei 11490, Taiwan; 6 Institute of Biomedical Sciences, National Sun Yat-sen University, Kaohsiung 80424, Taiwan; 7 Doctoral Degree Program in Marine Biotechnology, National Sun Yat-Sen University, Kaohsiung 80424, Taiwan; 8 College of Oceanology and Food Scienece, Quanzhou Normal University, Quanzhou 362000, China; johnjohnkings@163.com; 9 Department of Orthopedic Surgery, Pingtung Christian Hospital, Pingtung 90059, Taiwan; 10 Marine Biomedical Laboratory and Center for Translational Biopharmaceuticals, National Sun Yat-sen University, Kaohsiung 80424, Taiwan

**Keywords:** osteosarcoma, antimicrobial peptide, apoptosis, Fas/FasL pathway, mitochondria pathways

## Abstract

Osteosarcoma (OS) is a common malignant bone cancer. The relatively high density of a person’s bone structure means low permeability for drugs, and so finding drugs that can be more effective is important and should not be delayed. MSPs are marine antimicrobial peptides (AMP) and natural compounds extracted from Nile tilapia (*Oreochromis niloticus*). MSP-4 is a part of the AMPs series, with the advantage of having a molecular weight of about 2.7-kDa and anticancer effects, although the responsible anticancer mechanism is not very clear. The goal of this study is to determine the workings of the mechanism associated with apoptosis resulting from MSP-4 in osteosarcoma MG63 cells. The study showed that MSP-4 significantly induced apoptosis in MG63 cells, with Western blot indicating that MSP-4 induced this apoptosis through an intrinsic pathway and an extrinsic pathway. Thus, a pretreatment system with a particular inhibitor of Z-IETD-FMK (caspase-8 inhibitor) and Z-LEHD-FMK (caspase-9 inhibitor) significantly attenuated the cleavage of caspase-3 and prevented apoptosis. These observations indicate that low concentrations of MSP-4 can help induce the apoptosis of MG63 through a Fas/FasL- and mitochondria-mediated pathway and suggest a potentially innovative alternative to the treatment of human osteosarcoma.

## 1. Introduction

Osteosarcoma (OS) is a common malignant bone cancer, occurring most often in individuals under 20 years of age [1]. When there is no evidence of metastasis of osteosarcoma in patients there is a five-year survival rate of 60–70%, whereas the diagnosis of osteosarcoma metastasis in patients the survival rate of only 15–30% [2,3,4]. OS is most frequently present in the lower long bones, and the etiology of osteosarcoma so far is unknown. It may be related to genetic factors [5,6], and it may also be associated with chronic inflammation [7], radiation [8,9], and viral infection [10]. Malignant osteosarcoma cancer cells grow fast, easily expanding outward and invading normal tissue, often resulting in distant metastases even in the early stages of the disease. Osteosarcoma may easily recur after treatment, and, by finally causing the limbs to have dysfunction, the disease will end the patient’s life. Osteosarcoma is treated with high-dose individualized neo-adjuvant chemotherapy (also known as preoperative chemotherapy) and surgery [11,12], but chemotherapy’s effectiveness in osteosarcoma remains poor. The relatively high density of the bone structure means low permeability by the drugs used in chemotherapy, and so finding drugs that can be more effective is important and should not be delayed.

The process of programmed cell death, termed apoptosis, usually occurs during growth and aging and maintains the homeostatic mechanisms of cell populations in the biological tissue [13]. The process of cell death (apoptosis) is initiated by two pathways: the extrinsic (receptor-mediated apoptotic) and the intrinsic (mitochondria-mediated apoptotic); both are well established and represent the main mechanism of all mammalian cell apoptosis [14,15]. The absence of the two apoptotic pathways is associated with the carcinogenesis and pathogenesis of cancer cells [16,17]. The Fas group contains the Fas receptor (CD95/APO-1) and Fas ligand (FasL/CD95L). Fas activity must involve FasL; otherwise, agonistic anti-Fas antibody production of trimerization will lead to apoptosis. When the complex is trimerized and formed, the death-inducing signal complex (DISC) is initiated, eventually leading to the cleavage of caspase-8 and the subsequent effectors of caspase-3 in proteolytic protein with apoptosis. This is the extrinsic receptor (Fas/FasL)-mediated apoptotic pathway. Another intrinsic pathway, or the mitochondria-mediated apoptotic pathway, involves stimulating the caspase-8 cleavages Bid changes to t-Bid; however, the active cleavage of caspase-9 occurs through the mitochondrial release of cytochrome C protein. Eventually, the stimulated caspase-9 cleaves and activates the caspase-3 effectors with subsequent apoptosis [13,18,19].

MSPs are marine antimicrobial peptides (AMP) and natural compounds extracted from Nile tilapia (*Oreochromis niloticus*) because they can resist pathogen infections, and so they have been the focus of research on new antibiotics [20,21]. Although they usually destroy the cell membrane of the target cell directly, the action mechanism remains the subject of much scientific debate. However, their function is mainly to help the body fight against harmful substances within the body [22], interfere with bacterial proliferation [23], be a anti-nociceptor [24], and promote wound healing [25]. Recently, MSPs were also found to be useful in the treatment of liver cancer [26], cervical cancer [27], and fibrosarcoma [28]. MSP-4 peptide was synthesized utilizing the solid-phase method of Fmoc chemistry, and crude MSP-4 peptide was extracted, lyophilized, and purified by reversed-phase high-performance liquid chromatography (HPLC). The MSP-4 peptides’ molecular weight and purity were verified by mass spectrometry and HPLC. Synthetic peptides with over 95% purity were used in the experiment [29]. MSP-4 is one of the AMPs series, composed of about 25 amino acids and the amphiphilic α-helical cationic peptides structure, and has the advantage of having a molecular weight of about 2.7 kDa. The current research paper shows that MSP-4 can inhibit bacterial proliferation [20] and aid in immune regulation [22] and in the treatment of wounds [30] and breast cancer [31]. For other cancers, MSP-4’s effects and the workings of the mechanism are not very clear. This study aims to determine the mechanism associated with apoptosis resulting from the addition of MSP-4 to osteosarcoma MG63 cells.

## 2. Results

### 2.1. MSP-4 Decreased the Cell Viability of Osteosarcoma MG63 Cells

**Figure 1:**
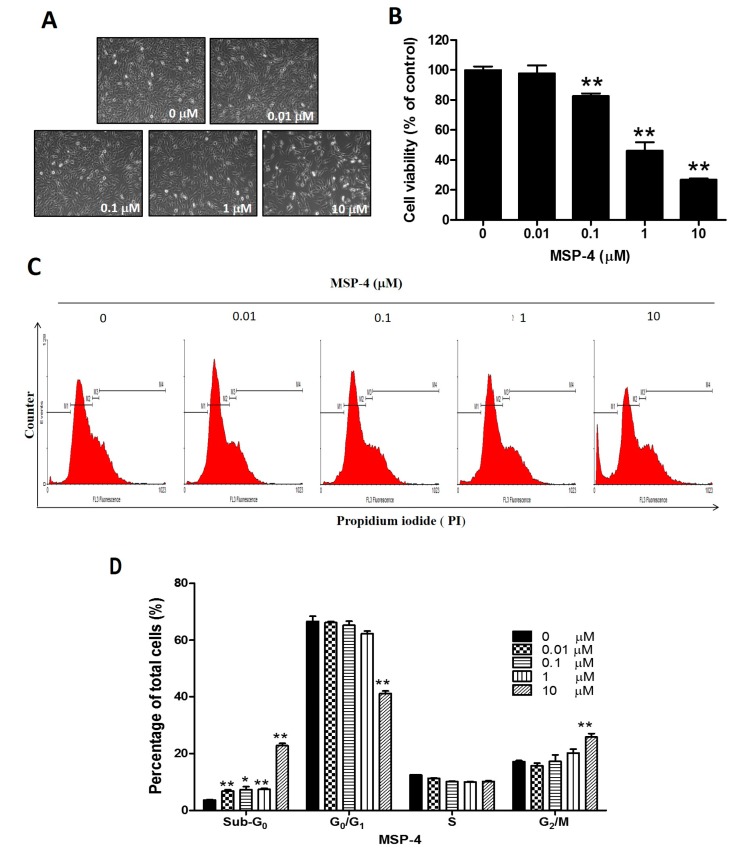
Effect of MSP-4 on the cell viability and cell cycle of MG63 cells. (**A**) MG63 cells were treated with the indicated various concentrations of MSP-4 for 24 h and photographed by phase-contrast microscopy at magnification (200×). (**B**) MTT assay was performed to measure cell viability. Cell viability (%) was expressed as a percentage compared to the untreated cells. The results were expressed as mean ± SEM (*n* = 6) of three independent experiments. (**C**) Cell-cycle analysis of MG63 cells treated with MSP-4 was conducted by flow cytometry with propidium iodide (PI). Cells were treated with the indicated concentrations of MSP-4 for 24 h. Cell-cycle distribution of PI-labeled cells was analyzed by flow cytometric analysis. The peaks in the illustration correspond to the apoptotic (Sub-G_0_), G_1_/G_0_, S, and G_2_/M phases of the cell cycle. (**D**) The histogram shows the percentages of cells in each phase of the cell cycle. The results are expressed as mean ± SEM (*n* = 3) of three independent experiments. The significance was determined by Student’s *t*-test * *p* < 0.05 and ** *p* < 0.01, compared with untreated cells.

Adding the MSP-4 drug to cultured media restrains the cell viability of human osteosarcoma cells in a d various dose-dependent manner. Figure 1A shows the cells’ morphology. MG63 cells with various concentrations of MSP-4 for 24 h were washed once and stained with MTT (3-(4,5-dimethylthiazol-2-yl)-2,5-diphenyltetrazolium bromide). This resulted in an approximate 70% decrease in proliferation, respectively, in MG63 cells compared to the vehicle controls. At concentrations of 0.1, 1, and 10 μM doses of MSP-4, cell viability was significantly reduced to 82.50 ± 3.63%, 46.16 ± 11.46%, and 26.78 ± 1.75% of the control level, respectively (Figure 1B). The MG63 cells exhibited a similar median lethal effective dose (LD_50_) to the MSP-4 at about 1 μM. These results suggest that MSP-4 can disturb the viability of the osteosarcoma cells.

### 2.2. MSP-4 Induced Apoptotic Cell Cycle Arrest in MG63 Cells

Measuring the DNA content of a variety of cells is a well-established method for monitoring the cell cycle and proliferation conditions. Therefore, when based on DNA content, the cell cycle is described by referring to the sub-G_0_, G_0_/G_1_, S, and G_2_/M phases. MSP-4-induced cell-growth inhibition in vitro could, in part, result from the modulation of the cell-cycle progression. To test this, MG63 cells treated with 0, 0.01, 0.1, 1, and 10 μM of MSP-4 for 24 h were stained with PI-containing RNase A and subjected to flow cytometry analysis. It was observed that MSP-4 arrested MG63 cells at the sub-G_0_ phase in a dose-dependent manner (Figure 1C). At concentrations of 0.01, 0.1, 1, and 10 μM doses of MSP-4, the sub-G_0_ population was significantly enhanced to 6.84 ± 0.86%, 7.32 ± 2.11%, 7.46 ± 0.75%, and 12.98 ± 2.05%, which indicated apoptotic cells, as compared to the untreated group (3.73 ± 0.24%). In the non-apoptotic population, the portion of cells in the G_0_/G_1_ phase decreased at a higher MPS-4 concentration (control, 0 μM: 66.64 ± 3.54%; 0.01 μM: 66.12 ± 0.90%; 0.1 μM: 65.22 ± 2.92%; 1 μM: 62.29 ± 1.78%; 10 μM: 50.62 ± 1.91%) with no effect on cells in the S phase, and the G_2_/M phase increased at a higher MPS-4 concentration (control, 0 μM: 17.17 ± 0.83%; 0.01 μM: 15.72 ± 1.95%; 0.1 μM: 17.29 ± 4.56%; 1 μM: 20.24 ± 2.73%; 10 μM: 26.53 ± 2.56%), respectively (Figure 1D). These results suggest that MSP-4 can induce cell-cycle arrest in the G_2_/M phase and increase the apoptotic cell phase (sub-G_0_) in osteosarcoma (MG63) cells in a dose-dependent manner.

### 2.3. Effect of Apoptosis by MSP-4 in MG63 Cells

**Figure 2:**
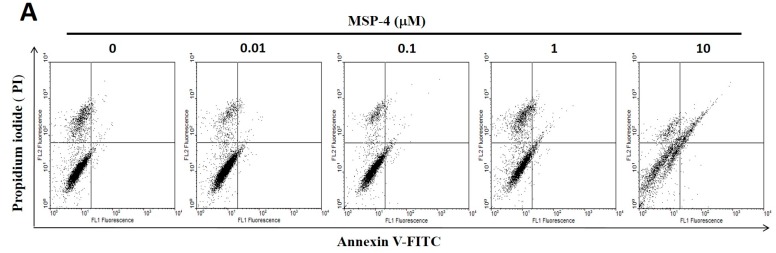

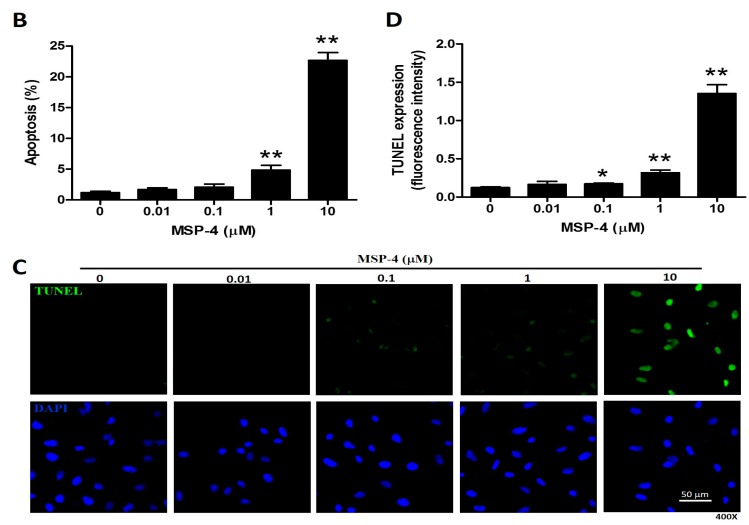
Apoptosis of MG63 cells treated with MSP-4 detected by flow-cytometry with annexin V-FITC/propidium iodide staining, as well as immunofluorescence TUNEL staining. (**A**) MG63 cells treated with MSP-4 for 24 h are shown with representative dot plots from FITC-conjugated annexin V (green color) and PI staining (red color). Cells in the lower-left quadrant (Annexin V-FITC −/PI −) are visible; early apoptosis was present in the lower-right quadrant (Annexin V-FITC +/PI −); late apoptosis was present in the upper-right quadrants (Annexin V-FITC +/PI +). (**B**) The percentage of apoptosis (early and late apoptoses) from flow cytometric analysis for 24 h was assessed. Values are mean ± SEM of three independent experiments in duplicate. (**C**) Immunofluorescence shows apoptotic MG63 cells marked by TUNEL assay with an increasing concentration of MSP-4 treatment for 24 h. The cell DNA/nuclei were stained using DAPI and visualized under a laser confocal microscope (400×). (**D**) TUNEL stain of MG63 cells in comparison with untreated cells markedly increased with the treatment of MSP-4. Data were calculated from 100–300 cells randomly selected for each treatment group. Relative quantification of the intensity of fluorescence was determined with the use of ImageJ software. Data were obtained from three independent experiments, and results were expressed as mean ± SEM. The significance was determined by Student’s *t*-test * *p* < 0.05 and ** *p* < 0.01 and compared with untreated cells.

It is well known that cell-toxicity effects are associated simultaneously with both intrinsic and extrinsic stimulations that lead to apoptosis. In order to confirm that MSP-4 induced apoptosis, we next determined that the cells displayed differential sensitivity to MSP-4-induced apoptosis through annexin V-FITC and PI (propidium iodide) double staining kit and TUNEL (In Situ Cell Death Detection Kit, Fluorescein) staining kit. As demonstrated in Figure 2A, MSP-4 did induce a higher level of apoptosis in MG63 cells, as expressed by annexin V/PI double stain and a flow cytometric assessment. At concentrations of 1 and 10 μM doses of MSP-4, the cell apoptotic rates significantly increased to 4.86 ± 1.52% and 12.65 ± 2.57% of the control level (1.15 ± 0.53%), respectively (Figure 2B). Using TUNEL (green color) staining to detect apoptotic cells and DAPI (4′,6-diamidino-2-phenylindole, blue color) staining to detect all nuclei and DNA fragmentation, which is the hallmark of apoptosis, was introduced to further analyze MG63 cells treated with MSP-4. As demonstrated in Figure 2C, treatment with MSP-4 induced a higher level of DNA fragmentation in MG63 cells, as revealed by immunofluorescence analysis. At concentrations of 0.1, 1, and 10 μM doses of MSP-4, the cell TUNEL-positive stain average of one-cell fluorescence intensity (green) significantly increased to 0.17 ± 0.22, 0.32 ± 0.07, and 1.35 ± 0.23 of the control level (0.12 ± 0.03), respectively (Figure 2D). In summary, these data showed that the apoptosis in MG63 cells was enhanced in response to MSP-4 treatment.

### 2.4. Effects of MSP-4 on the Protein Expression of the Death Receptor Pathway

**Figure 3:**
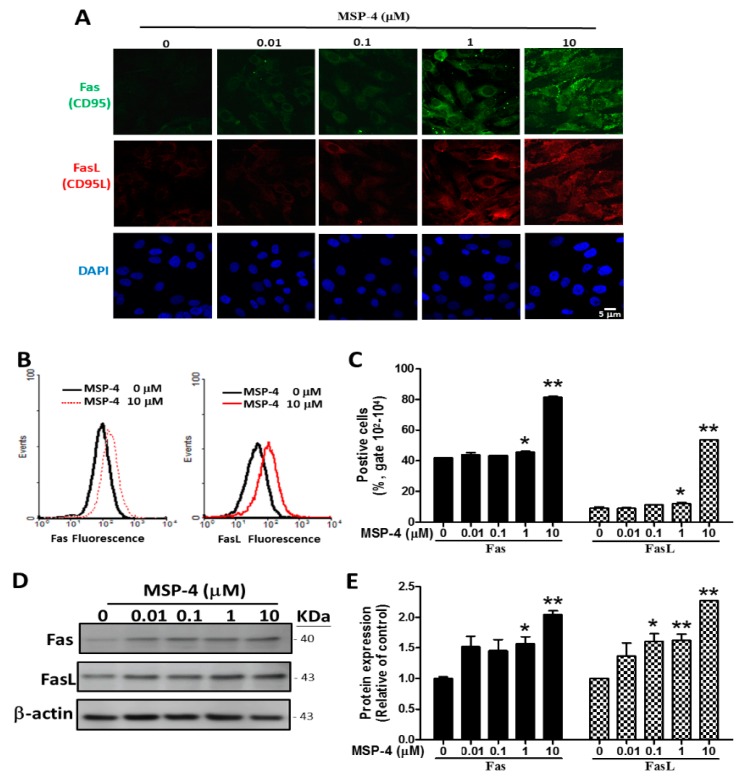
MSP-4 induced the Fas (CD95) and FasL (CD95L) expressions in MG63 cells. Fas and FasL protein expressions of MG63 cells were treated with MSP-4 as detected by immunofluorescence double staining, flow cytometry staining, and Western blot analysis. (**A**) Cells were pretreated with a dose-dependent of MSP-4 for 24 h and incubated with Fas or FasL antibodies for 1 h. They were then stained with Alexa 488- or 546-conjugated secondary antibodies (green or red). The cell DNA/nuclei were stained using DAPI (blue) and visualized under a laser confocal fluorescence microscope (1000×). Scale bar is 5 µM. (**B**) Flow cytometry analysis of surface FasL and Fas receptors. MG63 cells, treated in the absence and presence of 10 μM MSP-4 for 24 h, were incubated with Fas or FasL antibodies one hour before harvest and then stained with Alexa 488- or 546-conjugated secondary antibodies (green or red). The histograms of FL1 (Fas) fluorescence (Left) and FL2 (FasL) fluorescence (Right) shifted to the right as the MSP-4 dose escalated. (**C**) Quantification of MSP-4-induced production of Fas and FasL positive cell percentages by flow cytometry. Data were obtained from three independent experiments, and results were expressed as mean ± SEM. (**D**) Western blot analysis of surface FasL and Fas receptor. Here, -actin was detected as an internal control. (**E**) Blots band were analyzed using ImageJ software. Data were normalized using the β-actin level and expressed as a normalization of the control. Data (mean ± SEM) were representative of at least three independent experiments. The significance was determined by Student’s *t*-test * *p* < 0.05 and ** *p* < 0.01 and compared with untreated cells.

FasL is involved in the cell surface of the death receptor Fas (CD95) trimerization to elicit extrinsic apoptotic pathways [32]. In order to further elucidate the molecular mechanism of MSP-4-induced apoptosis in MG63 cells, we examined the protein expression of the Fas receptor/ligand by immunofluorescence, flow cytometry, and Western blot analysis. The protein expressions of both the Fas receptor and the ligand were induced by MSP-4 in MG63 cells in a dose-dependent manner. We demonstrated this by immunofluorescence double-stain analysis as shown in Figure 3A. However, the Fas receptor (green) and the ligand (red) were triggered much more prominently by the treatment concentrations of 1 and 10 μM of MSP-4 in the MG63 cells, whereas DAPI (blue) indicated a nuclear stain. As illustrated in Figure 3B, flow cytometry analysis of the Fas and FasL stains in the MG63 cells exposed to 0, 0.01, 0.1, 1, and 10 μM of MSP-4 results in the Fas (left) and FasL (right) flow histogram overlay figures at 10 μM of MSP-4 at the forward right move. At concentrations of 1 and 10 μM doses of MSP-4, the Fas statistics gates 10^2^–10^4^ significantly increased to 45.74 ± 1.28% and 81.52 ± 1.21% of the control level (41.87 ± 0.61%); however, the FasL statistics gates 10^2^–10^4^ significantly increased to 12.22 ± 1.27% and 53.72 ± 0.11% of the control level (9.32 ± 1.53%), respectively (Figure 3C). We used Western blot data again to confirm that Fas and FasL were induced by the treatment of MSP-4 in MG63 cells. Figure 3D shows the Western blot protein band profile with -actin as an internal control. Moreover, Western blot analysis revealed that MSP-4 treatment nearly doubled the Fas and FasL levels over the control (*p* < 0.01; Figure 3E). These observations suggest that MSP-4 can elevate Fas receptor/ligand levels and acquire sensitivity to Fas-apoptosis cell death in MG63 cells.

### 2.5. Effects of MSP-4 on the Protein Expressions of the Intrinsic Pathway

**Figure 4:**
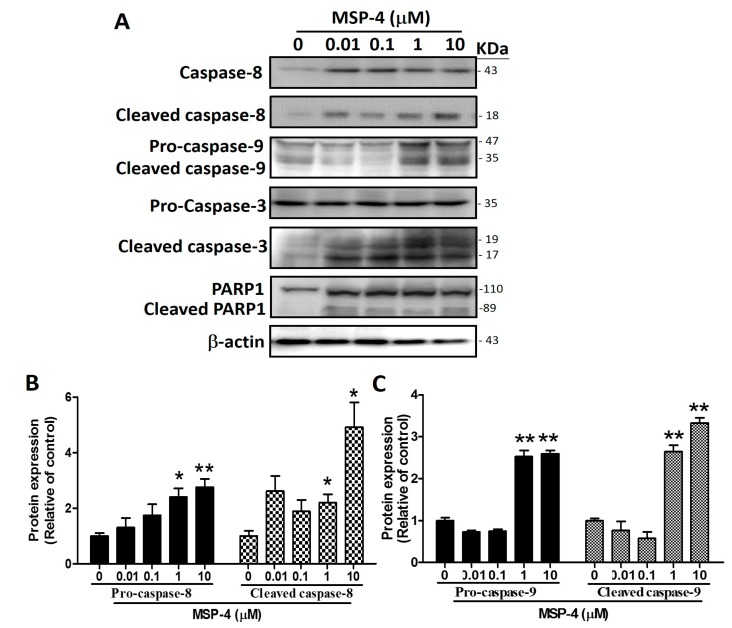

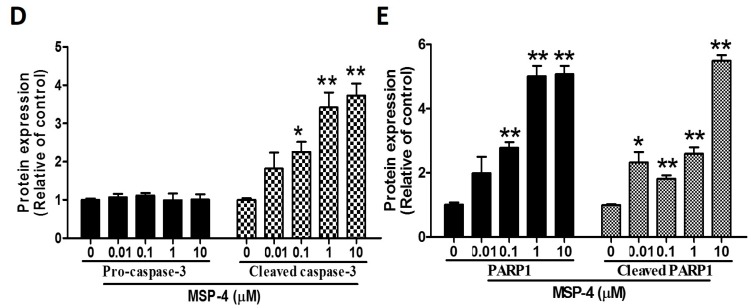
The effects of apoptosis-related protein expression in MG63 cells. (**A**) Cells were treated with different concentrations of MSP-4 for 24 h. Cell lysates were subjected to Western blot analysis using antibodies, including procaspase-3, cleaved caspase-3, caspase-8, cleaved caspase-8, caspase-9, PARP1, and -actin antibodies. Here, actin was detected as an internal control. The caspase-8 and cleaved caspase-8 (**B**), procaspase-9 and cleaved caspase-9 (**C**), procaspase-3 and cleaved caspase-3 (**D**), and PARP1 and cleaved PARP1 (**E**) protein levels were quantified by ImageJ software and normalized with that of the -actin level and expressed as a normalization of the control. Data (mean ± SEM) were representative of at least three independent experiments. The significance was determined by Student’s *t*-test * *p* < 0.05 and ** *p* < 0.01 and compared with untreated cells.

In order to carry out apoptosis, cells need to activate the caspase family [33]. Caspase-8 and -9 as initiators were activated by their own processing and by cutting downstream caspase-3 to activate the dimeric form of caspase-3 as the executor of apoptosis. To verify whether MSP-4 induces activation of these caspases, MG63 cells were exposed to MSP-4 at various concentrations, and the protein levels and cleaved form of caspases-3, -8, and -9 were examined by Western blot analysis. MSP-4 induced activation of caspase-8 (43 kDa) into cleaved caspase-8 (18 kDa), activation of procaspase-9 (47 kDa) into cleaved caspase-9 (35 kDa), and cleavage of procaspase-3 (32 kDa) into the active dimeric form of cleaved caspase-3 (19 and 17 kDa), respectively (Figure 4A). The PARP cleavage is due to the activation of caspase-3, which is also one of the characteristics of apoptosis, with cleavage of the PARP protein into 110 kDa (pro-form) and 89 kDa (cleaved form) fragments done through active caspase-3. Figure 4 shows that PARP1 was cleaved into an 89 kDa fragment after treatment of MSP-4 in a dose-dependent manner. As Western blot analysis revealed, treatment by exposure to MSP-4 (0–10 μM) for 24 h significantly elevated the caspase-8 and cleaved caspase-8 levels by about a 3- to 5-fold increment over the control (*p* < 0.05; Figure 4B). It also nearly tripled the procaspase-9 and cleaved caspase-9 levels over the control (*p* < 0.01; Figure 4C). The procaspase-3 level was unaffected, but the treatment cleaved the caspase-3 protein level by about a 3.5-fold increment over the control (*p* < 0.05; Figure 4D). PARP1 and the cleaved PARP1 level showed about a 5.5-fold increment over the control (*p* < 0.05; Figure 4E). All protein normalizations used -actin. These observations strongly indicate that MSP-4 induced apoptosis in MG63 cells via the mitochondria-dependent (intrinsic) signaling pathway.

### 2.6. Effects of MSP-4 on the Protein Expressions of the Bcl-2 Family

**Figure 5:**
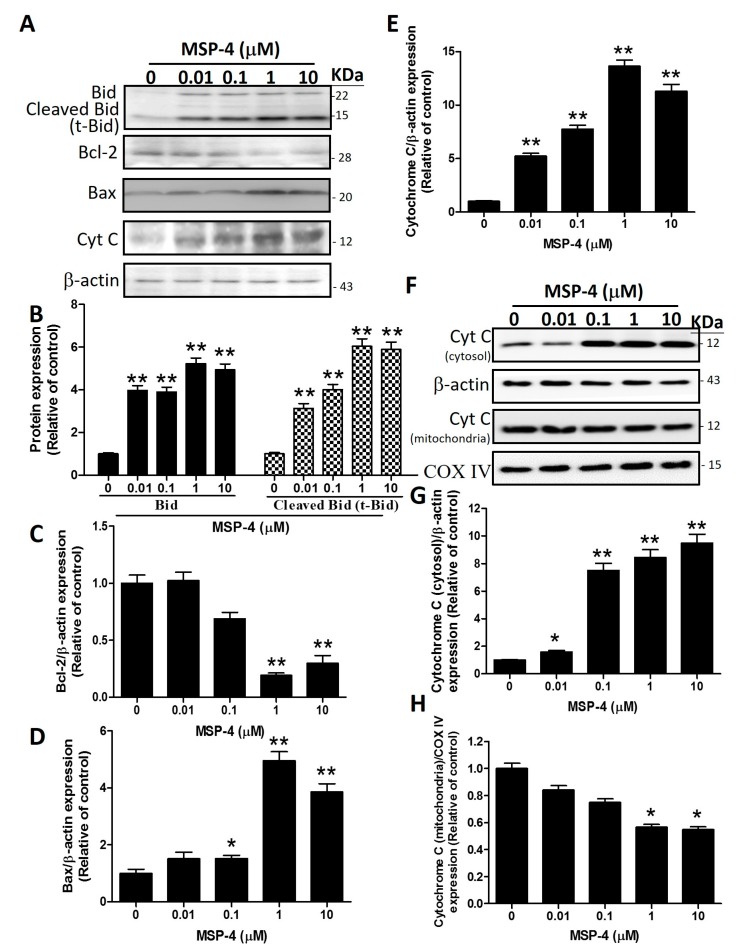
MSP-4 induced mitochondrial-related protein expression in MG63 cells. (**A**) Cells were treated with the indicated concentrations of MSP-4 for 24 h. Cell lysates were subjected to Western blot analysis using antibodies, including Bax, Bcl-2, Bid, cytochrome C, and -actin antibodies. Here, -actin was detected as the internal control. The Bid and cleaved Bid (t-Bid) (**B**), Bcl-2 (**C**), Bax (**D**), and cytochrome C (**E**) protein levels were quantified by ImageJ software and normalized with that of the -actin level and expressed as a normalization of the control. (**F**) The mitochondria/cytosol kits were isolated mitochondria and cytosol protein and then with Western blot analysis using antibodies, including COX IV, cytochrome C, and -actin antibodies; -actin and COX IV were detected as the internal control for cytosol and mitochondrial. Cytochrome C (cytosol) (**G**) and cytochrome C (mitochondria) (**H**) protein levels were quantified by ImageJ software and normalized with that of the -actin and COX IV levels and expressed as a normalization of the control. Data (mean ± SEM) were representative of at least three independent experiments. The significance was determined by Student’s *t*-test * *p* < 0.05 and ** *p* < 0.01 and compared with untreated cells.

Western blot analysis is a common method to analyze the expression of Bcl-2 family protein. This method was used to further investigate the molecular mechanism of MSP-4-induced apoptosis in MG63 cells in the Bcl-2 protein family. The Bcl-2 family, including pro-apoptotic proteins (Bid, Bax and Bak) and anti-apoptotic (Bcl-2 and Bcl-xL) proteins, makes up critical regulators of the mitochondria-mediated pathways for modulating the permeabilization of mitochondrial membranes [34]. MSP-4 treatment led to the downregulation of anti-apoptotic protein expression such as Bcl-2, while pro-apoptotic proteins**,** including Bax and Bid, were upregulated in a dose-dependent manner (Figure 5). However, Western blot analysis revealed that with exposure to MSP-4 (0–10 μM) in MG63 cells for 24 h, MSP-4 treatment significantly elevated the Bid and cleaved the Bid level by about a 5-fold increment over the control (*p* < 0.01; Figure 5B), the Bax level by about a 5-fold increment over the control (*p* < 0.01; Figure 5D), and the Bcl-2 protein level by about a 3.5-fold decrement over the control (*p* < 0.05; Figure 5C). Cytochrome C release from mitochondria into the cytoplasm is an indicator of mitochondrial-dependent apoptosis pathways. As shown in Figure 5A,F, cytochrome C gradually increases in the cytoplasm/total cells and increases according to the concentration of MSP-4. Western blot band analysis of cytochrome C in MG63 cells exposed to 0–10 μM of MSP-4 showed the cytochrome C protein level at about a 9- to 14-fold increment over the control (*p* < 0.05; Figure 5E,G), with all protein normalization using -actin. The cytochrome C gradually decreases in the mitochondria of MSP-4 (1 and 10 μM), and Western blot band analysis of cytochrome C protein level shows about a half-fold decrease over the control (*p* < 0.05; Figure 5H), with protein normalization using COX IV (cytochrome c oxidase complex IV). These results suggest that apoptosis was induced by MSP-4 in MG63 cells via the Bcl-2 family and the mitochondria pathway.

### 2.7. Effect of Caspase-8 (Z-IETD-FMK) and -9 (Z-LEHD-FMK) Inhibitors on Reverse MSP-4-Induced Activation

**Figure 6:**
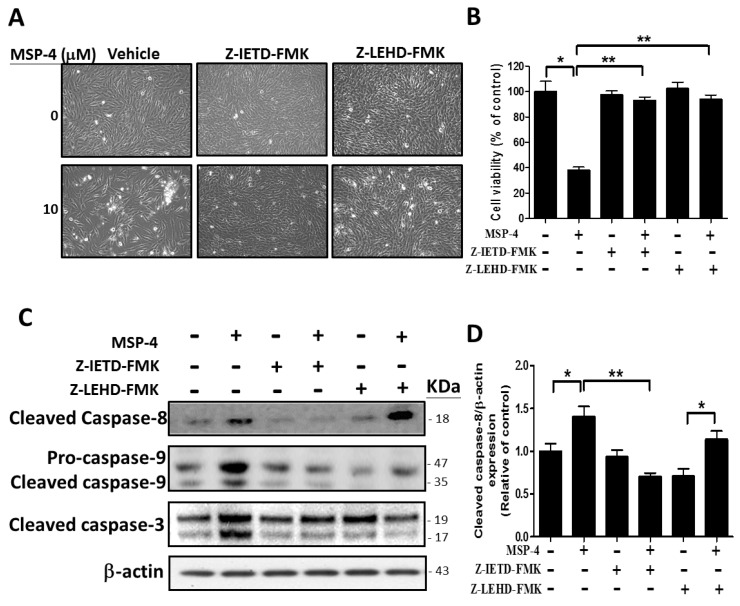

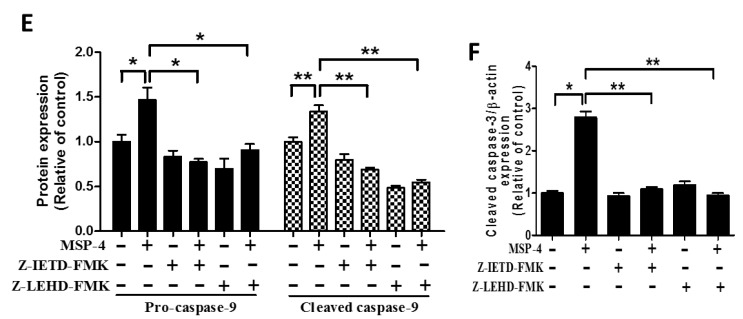
Caspase-8 and -9 inhibitors decreased apoptosis function and protein expression induced by MSP-4 in MG63 cells. (**A**) After prior incubation with caspase-8 or -9 inhibitors, including Z-IETD-FMK or Z-LEHD-FMK (10 μM), for 2 h, MG63 cells were treated without or with MSP-4 (10 μM) for an additional 24 h and were then photographed by phase contrast microscopy at 200× magnification. (**B**) The proliferation of MG63 cells was additionally determined by the MTT assay performed to measure cell viability. Cell viability (%) was expressed as a percentage compared to the untreated cells. The results were expressed as mean ± SEM (*n* = 3) of three independent experiments. (**C**) The representative Western blot analyses demonstrating the effects of the caspase-8 or -9 inhibitors, including Z-IETD-FMK or Z-LEHD-FMK, on caspase-8, caspase-9, and caspase-3 expressions in MG63 cells treated without or with MSP-4 (10 μM). Here, -actin was detected as the internal control. The cleaved caspase-8 (**D**), procaspase-9 and cleaved caspase-9 (**E**) and cleaved caspase-3 (**F**) protein levels were quantified by ImageJ software and normalized with that of the -actin level and expressed as a normalization of the control. Data (mean ± SEM) were representative of at least three independent experiments. The significance was determined by Student’s *t*-test * *p* < 0.05 and ** *p* < 0.01 and compared with untreated cells.

We demonstrated that MSP-4 did induce MG63 cells’ apoptosis. Next, the experiment analyzed the activation pattern of cell proliferation or caspase-8, -9, and -3 protein expression in the presence of specific inhibitors for each of the caspases-8 and -9 (Figure 6). Pretreatment of Z-IETD-FMK or Z-LEHD-FMK for 2 h while continuously adding MSP-4 drug in cultured media reversed the MG63 cells’ proliferation. This was photographed by phase contrast microscopy at 200× magnification. Figure 6A shows the cells’ morphology. We detected by MTT staining and statistics that Z-IETD-FMK or Z-LEHD-FMK partially reversed MSP-4-induced MG63 cell cytotoxicity (Figure 6B). To verify whether Z-IETD-FMK or Z-LEHD-FMK reversed the MSP-4-induced activation of these caspases, MG63 cells were exposed to Z-IETD-FMK or Z-LEHD-FMK for 2 h and then were simultaneously added with or without MSP-4. Western blot analysis was then used to examine the protein expression of caspases-3, -8, and -9, respectively (Figure 6C). Pretreatment of caspase-8 inhibitor, Z-IETD-FMK, inhibited the activation of cleaved caspase-8, procaspase-9, cleaved caspase-9, and cleaved caspase-3. Pretreatment of caspase-9 inhibitor, Z-LEHD-FMK, inhibited the activation of procaspase-9, cleaved caspase-9 (Figure 6E), and cleaved caspase-3 (Figure 6F), but it could not inhibit cleaved caspase-8 activation (Figure 6D). These data indicated that MSP-4 induced cytotoxicity and caspase-dependent apoptosis, but pretreatment with Z-IETD-FMK (caspase-8 inhibitor) or Z-LEHD-FMK (caspase-9 inhibitor) sharply suppressed the cells’ cytotoxicity and caspase expression in Western blots.

## 3. Discussion

The five-year survival rate of patients without metastatic disease is 60–70%, while the clinical outcomes for patients with metastatic disease are far worse, with a five-year survival rate of 15–30% reported. Resulting from the generation of immature bone cancer cells, OS is usually found at the end of longer bones, most often around the knees. The majority of those diagnosed with OS are under 20 years of age, as the disease is associated with the formation and growth of bone, and it seems to occur more frequently in males than in females. The etiology of osteosarcoma is so far unclear, but it may be related to genetic and familial factors. It may also be associated with chronic inflammation, radiation, viral infection, and alkylating agents [8,9,10,35]. Preoperative chemotherapy and postoperative chemotherapy, along with surgical resection of the tumor, are currently used to treat OS. Conventional treatment can cure 60–65% of primary cancer patients, but only 20–25% of patients with a recurrent disease. Therefore, new treatments and drugs are very necessary to improve the prognosis.

The pharmacological effects of biological activity on the treatment and prevention of cancer have increased dramatically over the past 10 years. Our laboratory, under Professor Jyh-Yih Chen at the Academia Sinica Institute, took five MSPs’ compounds of marine antimicrobial peptides (AMP) incorporating bioactive compounds from the Nile tilapia [20,21]. MSPs, small cysteine-rich molecules with about 25–80 amino acids, have an advantageously low molecular weight. They have also been found to inhibit cancer cells, such as breast cancer cells [31], cervical cancer cells [27,33,36], hepatoma cancer cells [26], and fibrosarcoma cells [28]. We screened five marine drugs (MSP-1, MSP-3, MSP-4, MSP-15, and MSP-33) for AMPs in osteosarcoma (MG63) cells (Figure 1B and Figure S1). We found that MSP-4 showed the most effective inhibition of MG63 cell proliferation, and a low dose disrupted cell growth, with IC50 resulting in about 1–5 M, but no influence on the proliferation on Hs68 cells (human normal fibroblast cell line) [30]. MSP-4 has also been reported in breast cancer studies to possess antitumor activity, with IC50 resulting in about 5.03 μM, which is similar to our experiments [31]. Our research showed that this difference in AMPs’ peptide produced distinctive anti-proliferative effects on human osteosarcoma cancer cell lines.

As a generalization, the possible and complex mechanisms of most of the many new compounds are considered to induce apoptosis and targeted apoptosis-signaling networks are becoming promising strategies for developing novel cancer therapeutic drugs [37,38,39]. The propidium iodide stain for cells by flow apparatus has been widely used for the judication of apoptosis in many experimental models. It is based on the experimental principle that apoptotic cells, along with other typical features, are characterized by DNA fragmentation and loss of nuclear DNA content. Using a table of binding and labeling DNA fluorochrome, such as PI, and then using flow cytometric analysis and identification of hypodiploid cells resulted in obtaining a rapid and precise evaluation of the cellular DNA content. Cell-cycle analysis using PI stain reveals the fragmentation of DNA (sub-G_0_) [40]. Our study with MSP-4 showed that the antitumor activity occurred through the apoptosis pathway. This was determined by morphology type and MTT stain showing quantitative cell viability, cell cycle by flow cytometric analysis for sub-G_0_, flow cytometric analysis for annexin V/PI stain showing the quantitative early and late apoptotic body, DNA fragmentation detection for the TUNEL stain, and activation of caspase-3 and the PARP cleaved form for Western blot analysis, respectively. However, many papers have reported that AMP and MSPs can induce apoptosis [27,41] and that necrosis [42,43] causes antitumor activity. Ting et al*.* reported that DNA fragmentation was not observed after MSP-4 treatment, indicating that MSP-4 does not induce apoptosis, but induces necrosis in breast cells [31]. In our experiment, the apoptosis production used different methods to demonstrate that cell death of osteosarcoma MG63 cells was also triggered by MSP-4.

Ting et al. reported that AMP-4 has defensive bacterial capabilities and belongs to the cationic antimicrobial peptides, allowing it to enter cancer cells through an anionic outer membrane [31]. However, the current study presents a different system that includes the Fas receptor/ligand. Fas [APO-l/CD95] is a transmembrane receptor expressed in many organisms. It belongs to the death receptor family and acts as the target of a cell-death-inducing antibody [44,45]. Apoptosis caused by extrinsic stimulation with a Fas receptor and a ligand-signaling system is an important step. When the Fas receptor-ligand is linked, Fas enters the cytoplasm to cause death-domain activation of apoptotic signaling, which interacts with the signal trans-conductors such as Fas-associated proteins associated with the death domain (FADD) and is downstream of the caspase cascade activation that plays a key role. The combination of Procaspase-8 with Fas-bound FADD resulted in the activation of caspase-8 to stimulate the caspase cascade, which subsequently resulted in cell breakdown, DNA degradation and eventual cell death [46]. This study showed and demonstrated for the first time that MSP-4 elicits apoptosis of MG63 cells through Fas receptor/ligand expression.

In most cases, the caspase-induced apoptotic responses are the central players in mammal cells [47]. The apoptotic caspase family is usually divided into two categories: initiator caspases (which may be further subdivided into intrinsic and external activators) including caspase-2, -8, and -9, and effector caspases, which include caspase-3 and -7. The most important function of all caspases in cells is to act as an enzyme that catalytically inactivates zymogenes. It is necessary to undergo proteolytic activation during the apoptotic process, and there is the little similarity in the N-peptide. The initiator caspases’ activation drives the effector caspases’ activation. Once the caspases are activated, the effector caspases are responsible for the majority of the cellular target proteolytic cleavage, which eventually leads to cell death [48]. Boulares et al. demonstrated that early breaks of nuclear protein poly(ADP-ribosyl)ation were required for apoptosis in various cell lines followed by the caspase-3-catalyzed cleavage of poly (ADP-ribose) polymerase (PARP) [49]. PARP was subsequently cleaved into 89 and 24 KDa fragments containing the enzyme active site and the DNA-binding domain, respectively. Our data showed that MSP-4-induced apoptosis proteins relied on caspase-8 protein that activated caspase-3 and PARP, which were finally expressed by anti-proliferation and DNA fragmentation in MG63 cells. Therefore, our study demonstrated that MSP-4 did induce apoptosis through caspase-8, -3, and PARP cleavage activation, which caused the operation of extrinsic mechanisms.

Mitochondrial membrane permeabilization (MMP) leads to the dissipation of mitochondrial membrane potential (∆ψm) and cell death [50]. Antimicrobial peptides can cause ∆ψm change by triggering apoptosis of cancer cells through mitochondrial membrane rupture [51]. The extrinsic apoptotic pathway can crosstalk with the intrinsic apoptotic pathway through the caspase-8-activated cleavage of “inactive” Bid (an alpha-helical, 22-kDa protein, a BH3-interacting domain death agonist of the Bcl-2 family of proteins) to produce a p15 Bid truncated fragment, called tBid, which translocates to the mitochondria [52]. This then triggers the release of mitochondrial-membrane-related proteins [53,54]. The p15 Bid (t-Bid) is able to target the mitochondrial site, where it turns into mitochondrial integral membrane protein. The mitochondrial pathway of apoptosis is dependent upon the Bcl-2 family, whose members have pro-apoptotic protein (Bid, Bax and Bak, etc.) and anti-apoptotic protein (Bcl-2 and Bcl-xL, etc.) functions, and regulates the permeability of the mitochondrial outer membrane to promote the effective release of apoptotic factors such as cytochrome C protein [55,56,57]. This cytochrome C (pro-apoptotic factor) is released from the inner mitochondrial membrane into the outer surface of the mitochondrial membrane at the early stage of apoptosis and binds to the cytosolic apoptotic protein, activating the apoptosis to promote the conversion of the protease caspase-9 with the transformation of caspase-3 and PARP in the active form. Apoptosis is hallmarked by a series of typical morphological features, such as shrinkage of the cell, nuclear fragmentation into membrane-bound apoptotic bodies, the breakdown of the cytoskeleton and phosphatidylserine exposure on the extracellular side of the plasma membrane, and rapid phagocytosis by neighboring cells [58,59]. Chen et al. showed that MSP-33 (Pardaxin) and tilapia (*Oreochromis mossambicus*) hepcidin TH2-3 treatment activates caspase-3 and cytochrome C in human fibrosarcoma HT-1080 cells [60,61].

The results herein showed that the MSP-4-peptide-induced apoptotic protein expression levels of anti-apoptotic protein such as Bcl-2 protein were downregulated, and the pro-apoptotic proteins Bax and t-Bid were upregulated in a dose-dependent manner. The release of cytochrome C from the mitochondrial membrane was also shown in MG63 cells with MSP-4 treatment at various doses. The protein levels of caspase-9 and -3 were continuously upregulated, and their active forms were also increased in association with the degradation of PARP. The MSP-4-induced apoptosis mechanism is activated by the intrinsic pathway (the mitochondria apoptotic pathway) and the extrinsic pathway (the receptor apoptotic pathway).

In the present study we demonstrated that increasing the caspase-8 and -9 levels influenced a reduction in cell viability, caused anti-proliferation, and induced apoptosis, with DNA being broken into small or separate parts and with a loss of nuclear DNA content. Caspase-8 and -9 are activated by the intrinsic pathway and the extrinsic pathway key point. These events partially promoted cell viability and blocked caspase-3, -8, and -9 protein expressions by caspase-8 (Z-IETD-FMK**)** and caspase-9 (Z-LEHD-FMK**)** inhibitors in MG63 cells. However, in the present study, in which caspase-8 protein was not blocked by caspase-9 inhibitor, caspase-8 and-9 from different pathways influenced apoptosis and finally influenced caspase-3 activation. In our study we showed that the intrinsic pathway (the mitochondria apoptotic pathway) and the extrinsic pathway (the receptor apoptotic pathway) activated the MSP-4-induced apoptosis mechanism. These results strongly support the use of MSP-4 in the development of drugs for human cancers, especially osteosarcoma and breast cancer, and provide encouragement that further preclinical and clinical studies will be very valuable. We hope that our continuous experimentation and research will bring about the use of MSP-4 in the treatment of human cancer, especially bone cancer, and that it will result in new beneficial opportunities.

**Figure 7:**
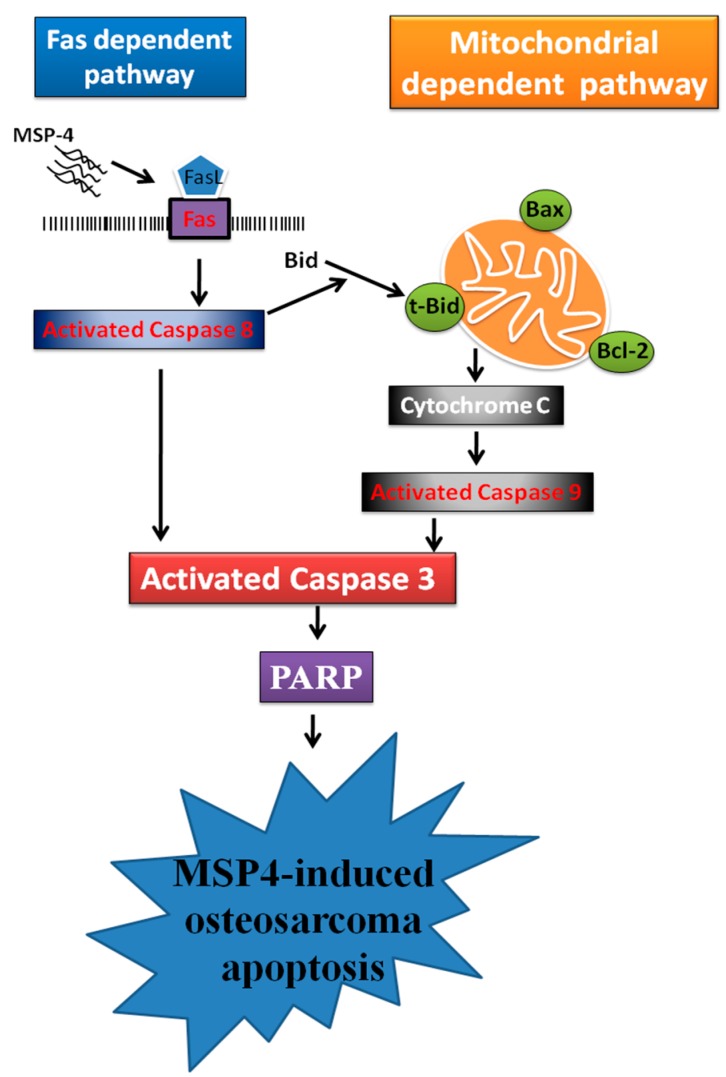
The hypothetical scheme for MSP4-mediated Fas/FasL and mitochondrial pathways in MG63 cells. The schematic diagram of MSP4 induces extrinsic and intrinsic apoptosis pathways. There are two major pathways involved in the process of caspase activation and apoptosis in osteosarcoma cells, including the extrinsic (death receptor Fas/FasL-mediated) pathway and the intrinsic (mitochondria-dependent) pathway.

Given the present results, we have summarized the apoptotic process signaling pathway based on the MSP-4-induced apoptosis mechanism in human osteosarcoma MG63 cells (Figure 7). Initially, MSP-4 triggers Fas ligand binding with its receptor and activates caspase-8 protein. The activated caspase-8 protein induced caspase-3 and PARP cleavage, linked to the cell-surface death-receptor apoptotic pathway. Moreover, the activated caspase-8 truncated Bid cleavage to tBid. Here, tBid, combined with the outer mitochondrial membrane, induced the Bcl-2 family, upregulated the pro-apoptotic Bax protein expression, and downregulated the anti-apoptotic Bcl-2 protein expression. It was able to continuously open the mitochondrial permeability pores of the outer mitochondrial membrane to release cytochrome C into the cytoplasm. The pro-apoptotic factor cytochrome C has its own tendency to enlarge the mitochondrial-dependent pathway to stimulate caspase-9 and downstream effector caspase-3 cleavage and to then carry on PARP cleavage to MSP-4-induced apoptosis of osteosarcoma MG63 cells. Taken together, our results indicate that the mechanism of the anti-tumor effect of MSP-4 is regulated by the external and internal pathways of apoptosis. Our findings also suggest the importance of MSP-4 antitumor activity, but not to affect normal cells, which may contribute to being an effective and strong therapeutic agent for osteosarcoma.

## 4. Materials and Methods

### 4.1. Reagents

TP-4 (FIHHIIGGLFSAGKAIHRLIRRRRR) was from Professor Jyh-Yih Chen’s laboratory at the Academia Sinica Institute. A stock solution of MSPs was prepared in phosphate buffered saline (PBS, pH = 7.2), protected from light, and stored at −20 °C. Annexin V/PI kit was purchased from Molecular Probes, Inc. (Eugene, OR, USA). An In Situ Cell Death Detection Kit (TUNEL) was purchased from Roche Applied Science (Mannheim, Germany). MTT, PI, RNase A, Z-IETD-FMK (caspase-8 inhibitor) and Z-LEHD-FMK (caspase-9 inhibitor) were purchased from Sigma-Aldrich Chemical Co. (St. Louis, MO, USA) and dissolved in DMSO or PBS.

### 4.2. Cell Culture

MG63 cells (ATCC^®^ CRL-1427™, Homo sapiens bone osteosarcoma) were cultured with -MEM medium (Minimum Essential Medium Eagle Alpha Modification; Gibco BRL, Rockville, MD, USA) containing 10% fetal bovine serum, 2 mM glutamine, 100 U/mL penicillin, and 100 µg/mL streptomycin (Gibco, Darmstadt, Germany) under a humidified atmosphere of 5% CO_2_ and 95% room air at 37 °C. For subculture, the cells were treated with trypsin-EDTA (Gibco, Darmstadt, Germany). The attached cells have the morphology of cobblestone when reaching confluence.

### 4.3. Cell Proliferation Assay

Cell viability levels were assessed useing a MTT stain assay following treatment with various concentrations of MSP-4 for 24 h. MTT drug was added to each well, and the plates were incubated at 37 °C for 2–4 h to allow MTT reduction through live cells dehydrogenases to an insoluble formazan product. Briefly, the cells were plated in triplicate at a density of 3 × 10^4^ cells per well in 24-well plates (Nunc, Roskilde, Denmark). Following overnight incubation, the cells were treated with MSP-4 at concentrations of 0, 0.01, 0.1, 1.0 and 10 μΜ for 24 h. Subsequently, the cells were observed under a microscope (Lecia Microsystems, Wetzlar, Germany), and the images were captured with a SPOT CCD RT-slider integrating camera (Diagnostic Instruments, Sterling Heights, MI, USA). Absorbance was recorded at 570 nm and was measured on an ELISA reader (Dynatech Laboratories, Chantilly, VA, USA). Cell viability was expressed as absorbance mean ± SEM.

### 4.4. Cell Cycle Analysis

MG63 cells were treated with MSP-4 at concentrations of 0, 0.01, 0.1, 1.0 and 10 μΜ for 24 h; they were then harvested, washed twice in PBS, and centrifuged. Cells were fixed with 100% ethanol at 4 °C for 2–4 h and were then stained with 50 μg/mL of PI and RNase A in PBS. The cell cycle was analyzed by a flow cytometer on a Beckman Coulter cytometer (Southfield,MI, USA) with the use of Cell Lab Quanta™ SC analysis software. A minimum of 20,000 cells per sample was analyzed.

### 4.5. Terminal Transferase-Mediated dUTP Nick-End Labeling (TUNEL) Assay

TUNEL assay was used to detect cells apoptosis and DNA fragmentation methods. MG63 cells were placed on cover slides with MSP-4 treatment at various concentrations of 0, 0.01, 0.1, 1.0 and 10 μΜ for 24 h and were fixed with 4% formaldehyde (pH = 7.2) for 5–10 min at 4 °C and washed with PBS. The stain was performed using an In Situ Cell Death Detection Kit Fluorescein according to the manufacturer’s protocol (Roche, Mannheim, Germany). Under immunofluorescent microscopy (Lecia Microsystems; Wetzlar, Germany), TUNEL positive cells were shown in green color. Moreover, those cells contained green fluorescence, as indicated by colocalization with DAPI (blue color) and by morphology. A laser confocal microscope was used with green fluorescence set at 488 nm and blue fluorescence set at 405 nm to view the slides. A SPOT CCD RT-slider integrating camera (Diagnostic Instruments Inc., Sterling Heights, MI, USA) was used to capture the images. The cells stained green were shown as apoptotic cells and blue indicated cell nuclear.

### 4.6. Annexin V/PI Staining

MG63 cells were treated with MSP-4 at various concentrations for 24 h then harvested, washed twice in PBS, and centrifuged. The 1 mL of the solution (6 × 10^5^ cells) was then transferred to a tube. Samples were processed for annexin V and propidium iodide (PI) label as per the manufacturer’s manual (BD Biosciences, San Jose, CA, USA). Cells were first resuspended in binding buffer and then labeled with fluorescence in order to detect early apoptotic, late apoptotic, and necrotic cells by adding 5 μL of annexin V-FITC and 5 μL of PI to each sample. Samples were gently vortex and placed at room temperature in the dark for 30 min. At the end of the incubation, 400 μL of 1× binding buffer was added to each sample, and the samples were analyzed using a Beckman Coulter flow cytometer with the use of Cell Lab Quanta™ SC analysis software. A minimum of 20,000 cells per sample was analyzed.

### 4.7. Fas and FasL Immunofluorescence Analysis

MG63 cells were treated with MSP-4 at various concentrations for 24 h and were fixed with 4% formaldehyde (pH = 7.2) for 5–10 min at 4 °C and washed with PBS. The cells were permeabilized using containing 1% bovine serum albumin in PBS, incubated with anti-Fas and anti-FasL for 1 h, and washed with PBS. Then the cells were incubated with Alexa-546- and 488-conjugated fluorescence antibodies (Thermo Fisher Scientific, Darmstadt, Germany) for 30–45 min at room temperature. The cell DNA/nuclei were stained using DAPI before mounting media. The slides were visualized under a laser confocal microscope (1000×, Lecia Microsystems, Wetzlar, Germany). A flow cytometry analysis was conducted to determine the surface FasL and Fas expressions in the MG63 cells. Cells treated with and without MSP-4 for 24 h, the cells were trypsinized and incubated with FasL and Fas first antibodies (1:100) in Flow Cytometry Staining Buffer (eBioscience, San Diego, CA, USA) at 37 °C for 1 h. After being washed twice with PBS, the cells were incubated with Alexa-488- and 546-conjugated secondary antibodies (1:200) at 37 °C for 1 h and resuspended in PBS for analysis using Beckman Coulter flow cytometry with the use of Cell Lab Quanta™ SC analysis software. A minimum of 10,000 cells per sample was analyzed.

### 4.8. Mitochondrial and Cytosol Isolation Methods

The Mitochondria/Cytosol isolation in the MG63 cell were isolated by Mitochondria/Cytosol Fractionation kit (BioVision, Inc., Milpitas, CA, USA) following the manufacture’s protocols and the “Western Blot Analysis” methods.

### 4.9. Western Blot Analysis

The cell lysates from MG63 cells were treated with MSP-4 at 0, 0.01, 0.1, 1.0 and 10 μΜ of concentrations for 24 h or they were pretreated with or without caspase-8 and -9 inhibitor (10 μM) for 2 h, then additional cells with or without treatment of MSP-4 (10 μΜ) for 24 h were obtained, and protein quantify concentrations were determined by the Bradford method (Bio-Rad, Hercules, CA, USA). After separation using 8–12% SDS-PAGE, they were transferred onto a polyvinylidene difluoride (PVDF) (Millipore, Bedford, MA, USA) membrane. The membrane was blocked with 5% non-fat milk and then incubated overnight at 4 °C with antibodies of anti-Fas, anti-FasL, anti-PARP1, anti-caspase-3, anti-cleaved caspase-3, anti-caspase-8, anti-caspase-9, anti-Bax, anti-Bcl-2, anti-cytochrome C, anti-Bid, anti-cleaved caspase-8 and COX IV (1:1000 dilution; Cell Signaling Technology) and anti-β-actin (1:50,000 dilution; Millipore) antibodies. Following conjugation of the secondary antibody with horseradish peroxidase at 37 °C for 1 h, the signals on the membrane were detected using enhanced chemiluminescence (ECL-kit, Millipore, Darmstadt, Germany). Photos were taken on the visualized bands using UVP BioChemi imaging (UVP LLC, Upland, CA, USA). Relative densitometry quantification of bands were performed using LabWorks 4.0 software (UVP LLC). A β-actin antibody was used to reprobe the PVDF membranes as a loading control.

### 4.10. Statistical Analysis of Studies

SPSS (Windows 13.0 version) was used for all statistical analysis data. The independent *t*-test on continuous variables showed the results as mean ± SEM. Results were analyzed by the *t*-test. ** *p* < 0.01 or * *p* < 0.05 was considered to be statistically significant.

## Supplementary Materials

The following are available online at www.mdpi.com/1660-3397/16/1/8/s1, Figure S1: Effect of MSP-1, MSP-3, MSP-15, and MSP-33 on cell viability of MG63 cells.
