# Supplementary material for: MSP-4, an Antimicrobial Peptide, Induces Apoptosis via Activation of Extrinsic Fas/FasL- and Intrinsic Mitochondria-Mediated Pathways in One Osteosarcoma Cell Line
Source: Mar Drugs. 2018 Jan 2;16(1):8. doi: 10.3390/md16010008 (PMC5793056; doi:10.3390/md16010008)
Supplement: Supplementary file 1 [file marinedrugs-16-00008-s001.pdf]

Supplementary Materials: MSP-4, Antimicrobial Peptide, Induces Apoptosis via Activation of Extrinsic Fas/FasL- and Intrinsic Mitochondria-Mediated Pathways in One Osteosarcoma Cell Line

Hsiao-Mei Kuo, Chung-Chih Tseng, Nan-Fu Chen, Ming-Hong Tai, Han-Chun Hung, Chien-Wei Feng, Shu-Yu Cheng, Shi-Ying Huang, Yen-Hsuan Jean, Zhi-Hong Wen

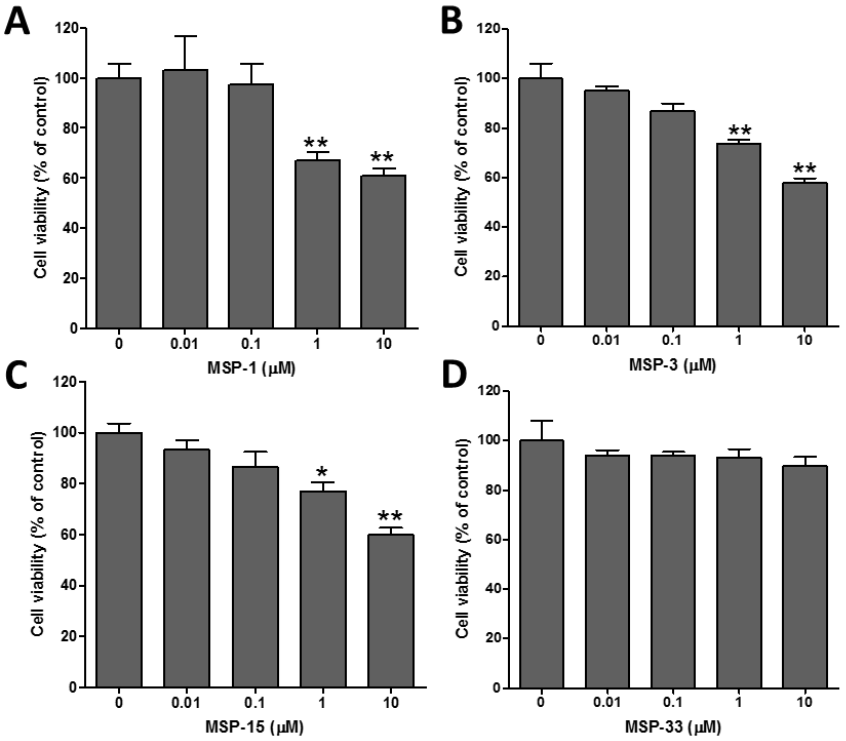

Figure S1. Effect of MSP-1, MSP-3, MSP-15, and MSP-33 on cell viability of MG63 cells. Cells were treated with an increasing concentration of MSP-1 (A), MSP-3 (B), MSP-15 (C) and MSP-33 (D) for 24 h, and then an MTT assay was performed to measure cell viability. Cell viability (%) was expressed as a percentage compared to the untreated cells. The results were expressed as mean ± SEM (n=6) of three independent experiments. The significance was determined by Student's t-test *P < 0.05, **P < 0.01, compared with untreated cells.
